# The Free State Collection for Anthropological Research (FS-CAR): a new contemporary identified skeletal collection in South Africa

**DOI:** 10.1007/s00414-023-03086-y

**Published:** 2023-09-18

**Authors:** Petra Maass

**Affiliations:** Department of Basic Medical Sciences, Faculty of Health Sciences, University of the Free State, 205 Nelson Mandela Drive, Park West, Bloemfontein, 9300 South Africa

**Keywords:** Forensic anthropology, Identified skeletal collection, Human skeletal biology, South Africa

## Abstract

Documented skeletal collections are valuable resources for anthropological studies aimed at reconstruction of the biological profile and examination of osteological trauma and pathology. The Free State Collection for Anthropological Research is a new, contemporary skeletal collection, based in central South Africa that has become available for such studies. This paper aims to provide an overview of the collection to encourage its future use in national and international research. The collection currently contains 64 female and 122 male skeletons of individuals that have died in the Free State province since the year 2000. Black individuals constitute 94.6% of the collection. Age-at-death ranges between 19 and 86 years, with an overall mean of 40 years. Year-of-birth ranges from 1927 to 1991. Tuberculosis (22.6%) and influenza/pneumonia (20.4%) are the most common cause of death for these individuals. Although the current demographic profile of the collection is skewed, new individuals are continuously being added. The collection offers several opportunities for anthropological research. The relatively young age-at-death and contemporary nature of the collection make it a useful tool for validation of existing methods for estimations of components of the biological profile. It can also be used in conjunction with other collections for the development of new methods for age and sex estimation and studies of trauma and disease manifestation of the skeleton.

## Introduction

Identified human skeletal collections are the cornerstone for research on human skeletal variation. Using these collections, anthropologists are able to develop new methods and test existing ones for estimations of components of the biological profile, which is of value for both biological anthropology and its application in forensic contexts [1–7]. Additionally, manifestation of pathology on these skeletal remains—especially when cause of death information is available—enables comparative paleopathology studies, while the presence of trauma and healing enables refining of macroscopic characteristic descriptions [4, 5, 7, 8].

Globally, many collections have been established since the twentieth century and are widely referenced in the literature [9]. Despite the benefits of these collections, some questions regarding the use and interpretation of data collected from these samples are still debated in the literature [8, 10]. Advances in imaging and 3D scanning technologies have also resulted in a shift toward a more virtual approach to anthropological analyses [11, 12]. However, cost and accessibility of these technological approaches, lack of agreement regarding standardization, and ethical concerns still limit their widespread use in many developing countries [12]. Often, use of such technologies is also not practical in forensic contexts where poorly funded government facilities are unable to capture data in the required formats, and antemortem data for comparison is unavailable for the majority of cases [13]. While the benefits of digitization are clear and access is improving, many support the continued use of skeletal collections for macroscopic analyses and use of advanced technologies, when available, as complementary [11, 12].

Examination of skeletal morphology in existing collections has shown variation both geographically and temporally [1]. This increases the value of geographically diverse skeletal collections to allow for examination of such variation and possible genetic and environmental influences thereon and perhaps even as the source for data to be included in the development of “global” standards for estimations of the biological profile [14, 15].

Several countries including the USA [16], Brazil [17], and European countries including Greece [18], Portugal [1, 2, 19], and Italy [4, 6] have benefitted from having multiple skeletal collections, allowing descriptions and comparisons of various population groups within and between countries. The number of collections in Latin America, Asia, and Africa is also increasing [9]. Currently, all of the skeletal collections on the African continent are located in South Africa [9], with documented collections housed at the University of Pretoria [20], University of the Witwatersrand [21], Stellenbosch University [22], and the University of Cape Town [23]. In 2017, another collection, the Free State Collection for Anthropological Research (FS-CAR), was established at the University of the Free State in Bloemfontein, South Africa.

The aim of this paper is to provide a detailed description of the composition of this new, contemporary cadaveric skeletal collection to encourage the use thereof for future national and international studies and collaborations.

## The Free State Collection for Anthropological Research

The Free State Collection for Anthropological Research (FS-CAR) contains skeletal remains of cadavers received by the department for use in medical training and research, in accordance with the National Health Act of South Africa. Cadavers are received as either self- or next-of-kin donations (“bequests”) or as “unclaimed” via government hospitals where they died without next-of-kin being located or being able to claim the remains within approximately 30 days after death. The department only accepts individuals that have died within 300 km of Bloemfontein and of natural causes (as determined by the attending health care practitioner). Following use of the cadavers in the medical dissection program, some are selected for inclusion in the skeletal collection, following cleaning and degreasing of the bones. Though individuals over 50 years of age were previously excluded from the collection, this stipulation has been removed, and the collection now accessions skeletal remains of any individual over 18 years old that has been received by the department since the year 2000.

For each individual in the FS-CAR, information such as sex, age, population affinity (as per the terminology of the latest national census [24]), year-of-birth, and nationality is available. This information is obtained from the official government-issued death certificate and other hospital records received with the remains upon receipt by the institution. Additionally, cause of death information is available for all individuals accessioned before 2014, as amendments to national legislation prohibit access to this information in subsequent years.

### Demographic compositions of the collection

The FS-CAR currently contains the skeletal remains of 186 individuals. Of these, 16 individuals are represented by complete skeletons and three individuals by partial postcranial elements only. The remaining 167 individuals have both cranial and postcranial elements, but more than 10% of their bones are absent. The most commonly absent elements are the mandible and ribs, which are usually cut during the cadaver dissection process, and less dense bones such as the sternomanubrium, which is often damaged during the maceration process. Overall, the bones are in excellent condition with only few alterations such as decortication of elements from older individuals and pencil markings and minor chipping of bone edges as a result of handling by students.

Though all individuals in the collection had last known addresses in the Free State province of South Africa, three foreign individuals are included in the collection—two Black females from neighboring Lesotho and one Black male from Ethiopia.

The composition of the FS-CAR according to sex and population affinity is shown in Table 1. Males currently outnumber females at a ratio of approximately 2:1. The majority of remains are of Black individuals (94.6%), with only nine White individuals (3 females; 6 males) and one Colored female. There are currently no Colored males or any Indian/Asian individuals in the collection.

**Table 1:** Composition of the Free State Collection for Anthropology Research according to sex and population affinity*

Population affinity	Sex				Total	
	Female		Male			
	*n*	%	*n*	%	*n*	%
Black	60	32.3	116	62.4	176	94.6
Colored	1	0.5	–	–	1	0.5
White	3	1.6	6	3.2	9	4.8
Total	64	34.4	122	65.6	186	100

^*^As categorized by the latest national census[24]

**Fig. 1:**
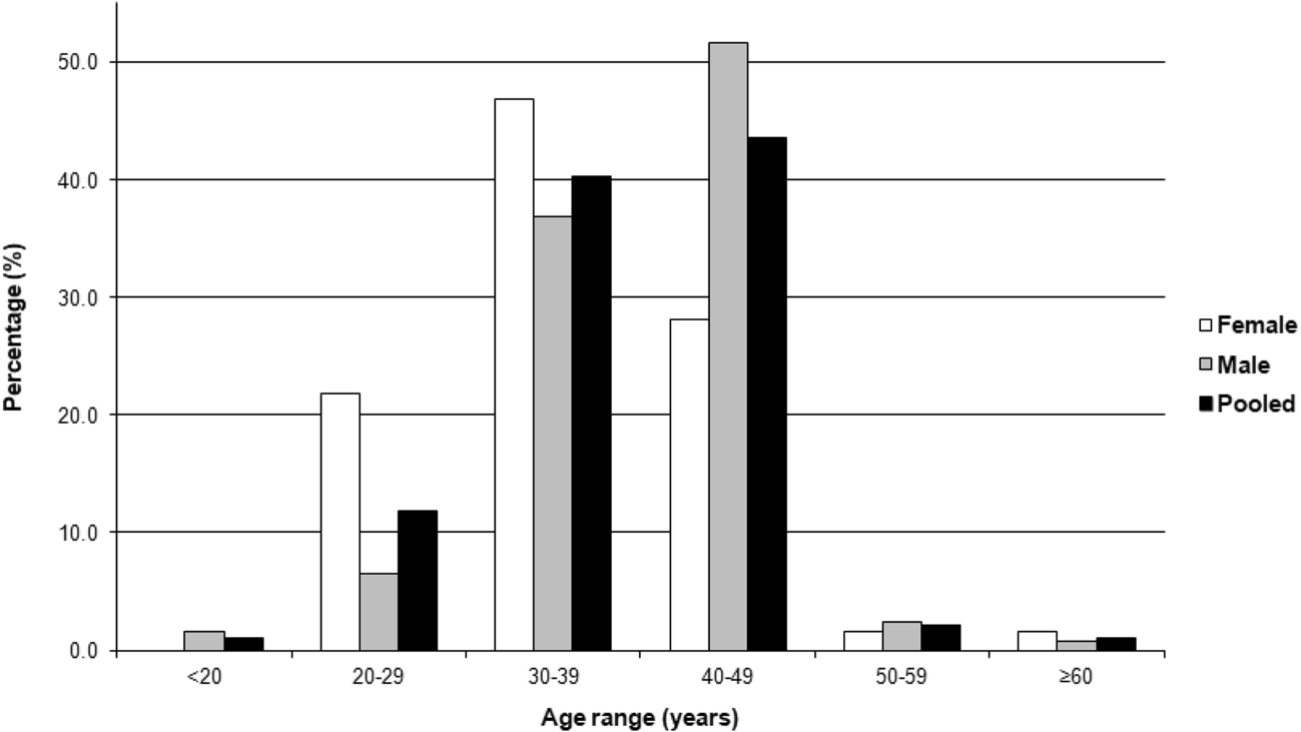
Percentage distribution of individuals of the Free State Collection for Anthropological Research in ten-year age ranges

**Fig. 2:**
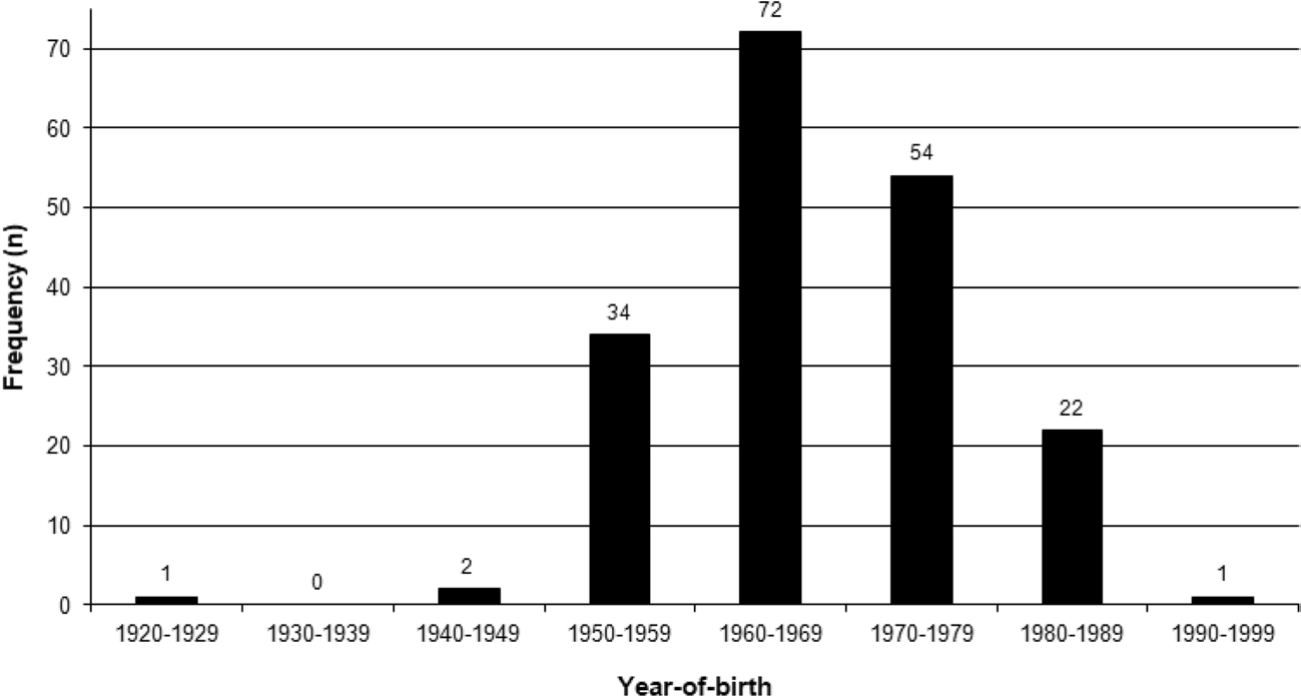
Distribution of year-of-birth of the Free State Collection for Anthropological Research

Age-at-death of individuals in the collection ranges from 19 to 86 years, with a mean of 40 years (Table 2). The distribution is, however, notably skewed (Fig. 1), with 83.9% of individuals being between the ages of 30 and 50 years. Mean ages of the male and female groups differ by only four years, though the age range of the female group is much larger, mostly due to the presence of one 86-year-old female. The year-of-birth of individuals in the collection ranges from 1927 to 1991, with the majority being born in the 1960s and 1970s (Fig. 2).

**Table 2:** Age distribution of the Free State Collection for Anthropological Research

	Age distribution (years)								Mean (range)
	< 20	20–29	30–39	40–49	50–59	60–69	70–79	80–89	
Overall	2	22	75	81	4	1	–	1	40 (19–86)
Female	–	14	30	18	1	–	–	1	37 (22–86)
Male	2	8	45	63	3	1	–	–	41 (19–64)
Black	2	21	71	78	3	1	–	–	39 (19–64)
Colored	–	–	1	–	–	–	–	–	36
White	–	1	3	3	1	–	–	1	47 (29–86)
Black female	–	14	27	18	1	–	–	–	37 (22–55)
Black male	–	2	7	44	60	2	1	–	41 (19–64)
Colored female	–	–	1	–	–	–	–	–	36
White female	–	–	2	–	–	–	–	1	53 (32–86)
White male	–	1	1	3	1	–	–	–	44 (29–57)

### Cause of death

Cause of death information is available for 158 individuals in the collection. The listed primary causes of death of these individuals were classified according to the ICD-10 [25] coding standards (Table 3). The leading cause of death classification was infectious and parasitic diseases (41.8%), with 42 (63.6%) of these individuals having died of tuberculosis. The second leading cause of death was diseases of the respiratory system (32.9%), of which the majority (*n* = 38; 73.1%) were due to influenza/pneumonia.

**Table 3:** International classification of diseases (ICD-10) causes of death for cadaveric remains in the Free State Collection for Anthropological Research

ICD-10 chapter		Pooled		Female		Male	
		*n*	%	*n*	%	*n*	%
I	Certain infectious and parasitic diseases	66	41.8	20	40.8	46	42.2
II	Neoplasms	3	1.9	2	4.1	1	0.9
III	Diseases of the blood and blood-forming organs and certain disorders involving the immune mechanism	1	0.6	–	–	1	0.9
IV	Endocrine, nutritional, and metabolic diseases	9	5.7	7	14.3	2	1.8
VI	Diseases of the nervous system	9	5.7	1	2.0	8	7.3
IX	Diseases of the circulatory system	6	3.8	1	2.0	5	4.6
X	Diseases of the respiratory system	52	32.9	15	30.6	37	33.9
XI	Diseases of the digestive system	1	0.6	–	–	1	0.9
XII	Diseases of the skin and subcutaneous tissue	1	0.6	–	–	1	0.9
XIV	Diseases of the genitourinary system	5	3.2	2	4.1	3	2.8
XVIII	Symptoms, signs, and abnormal clinical and laboratory findings not elsewhere classified	5	3.2	1	2.0	4	3.7

## Potential and contribution of the collection

The FS-CAR is the latest addition to identified human skeletal collections in South Africa, representing a modern collection of individuals that have died in the central parts of the country in the past 23 years. Despite being smaller than other South African collections, the FS-CAR shows some similarities to these collections. Firstly, similar overrepresentation of males and Black individuals has been noted, especially for the Pretoria Bone Collection and Raymond A Dart Collection [20, 21]. This skewed demographic profile reflects the influences of the stipulations of the National Health Act and the sociocultural aspects associated with donations. Black South Africans are reportedly less likely to donate their remains to science [26–28], which some ascribe to religious and cultural beliefs [20, 27, 29, 30]. The considerable number of Black males in South African collections is thus usually of unclaimed individuals who have migrated to the northern parts of the country for mining or agricultural work [31, 32]. Often when these individuals die, next-of-kin cannot be reached or are unable to afford claiming the body, resulting in the body being donated to medical schools. Though Black females also migrate for work, they tend to stay closer to and remain in closer contact with their families [32, 33], thus making return of the body to the family easier [20, 29].

The FS-CAR has a mean age-at-death of approximately 20 years younger than that of the other South African collections [20–23]. The sizable proportion of individuals 30 to 50 years old is attributed to previous departmental practices, where individuals over 50 years old were not macerated to obtain skeletal material, as it was expected that the bones would be too brittle for the process. This has recently been changed, and skeletal remains of all individuals over 18 years are now included in the collection. This will hopefully over time reduce the skewed age distribution of the collection, while adding more individuals in all adult age groups. Despite the age differences, the year-of-birth range of the FS-CAR places it as a chronological contemporary to the other middle to late twentieth century collections in the country [20–23].

The skewed demographic profile of the FS-CAR is currently a limiting factor in what studies are possible based on this collection alone. However, the younger ages of individuals in this collection, and the fact that the FS-CAR exclusively accessions individuals that have died in the twenty-first century, make this collection a useful tool for research into aspects such as secular trends or method validation studies of sex and age [34], especially when considered along with samples from the other South African collections [2, 6, 7, 20].

The other aspect in which the FS-CAR has research potential is in the availability of cause of death information. This information can be used to investigate and interpret pathological lesions on the bones or consequences of diseases such as stunted growth, which has both bioarcheological and forensic application [4, 7]. The leading underlying causes of death in the collection, tuberculosis and influenza/pneumonia, are among the leading provincial causes of death, especially for individuals in their thirties and forties [35]. This makes the FS-CAR useful for studies focused on the effects of these kinds of communicable diseases, as the more elderly collections elsewhere tend to have more noncommunicable diseases, such as neoplasms and cardiovascular conditions, as cause of death [22, 23, 26, 27]. In addition, several individuals in the FS-CAR show signs of nonfatal conditions (e.g., vertebral osteophytosis) and antemortem trauma (e.g., craniofacial trauma), which can be studied.

In only the first five years of operation, the FS-CAR remains have been utilized in two Master of Medical Science and eight Bachelor of Medical Science Honors research projects. Topics of these projects include developing regional standards for sex and stature estimation and evaluating age estimation accuracy based on transition analysis, as well as descriptive studies of craniofacial asymmetry, physiological stress indicators, craniofacial blunt force trauma, and degenerative disease in the vertebral column. Although the collection is currently relatively limited, it is slowly growing both in its size and potential for use in anthropological research studies.

It is hoped that the information provided in this paper will encourage use of the collection as a resource for future studies and collaborations. Interested researchers may contact the corresponding author/curator for more information regarding applications for access to the collection.

## Data Availability

The Free State Collection for Anthropological Research (FS-CAR) is available for research.
